# Investigating the sleep habits in individual and team-sport athletes using the Athlete Sleep Behavior Questionnaire and the Pittsburgh Sleep Quality Index

**DOI:** 10.5935/1984-0063.20210031

**Published:** 2022

**Authors:** Matthew W. Driller, Haresh Suppiah, David Rogerson, Alan Ruddock, Lachlan James, Adam Virgile

**Affiliations:** 1 La Trobe University, Sport and Exercise Science, School of Allied Health, Human Services and Sport - Melbourne - Victoria - Australia.; 2 Sheffield Hallam University, Sport and Physical Activity Research Centre - Sheffield - England.; 3 University of Vermont, Department of Nursing and Health Sciences - Burlington - Vermont - United States.

**Keywords:** Sleep hygiene, Exercise, Circadian Rhythm, Caffeine

## Abstract

**Objectives:**

There is limited large-scale data on maladaptive sleep practices in elite adult athletes and their influence on sleep characteristics. This study aimed to identify differences in sleep behaviours between individual and team-sport athletes using two sleep questionnaires.

**Material and Methods:**

407 (237 male, 170 female) elite adult athletes across sixteen sports (9 individual-sports, 7 team-sports) completed the Athlete Sleep Behavior Questionnaire (ASBQ) and the Pittsburgh Sleep Quality Index (PSQI).

**Results:**

Individual-sport athletes reported greater total sleep time and higher sleep efficiency than team-sport athletes (*p*<0.05, *d*=0.28-0.29). There were no differences between global scores for the PSQI, however, there was a difference between global scores for the ASBQ as well as multiple individual items from both questionnaires (p<0.05), indicating poorer behaviours in team-sport athletes.

**Discussion:**

Team-sport athletes displayed more maladaptive pre-sleep behaviours and poorer sleep characteristics than individual-sport athletes.

## INTRODUCTION

The description and influence of sleep quantity and quality in elite athletes has seen an exponential increase in the research literature over the last decade, drawing considerable attention to the practical application of promoting sleep practices in the athlete setting^1,2^. This is primarily due to the mounting evidence that athletes may face many unique challenges to obtaining the recommended amount of restful sleep per night. While the exact duration of sleep required by athletes is likely to be highly individual, previous researchers have recommended that for adults, seven to nine hours of sleep per night is recommended, with less sleep than this considered suboptimal, and likely to compromise health, well-being, and performance^3^. Therefore, given the increased demand for both physiological and psychological recovery in athletes, the optimal sleep duration is likely to be towards the top end of this recommended range^4^. Physiological, psychological and scheduling factors have all been identified as issues that could compromise sleep quality and quantity in athletes^5-7^, and these factors are likely to differ between individual and team-sport athletes.

Individual-sport athletes are more likely to have greater control over their daily training schedules and routines, with more irregular competitions schedules, whereas team-sport athletes may have less control over their training schedules and more regular week-to-week competition formats. There are also numerous personality traits that are associated with individual and team-sport athletes that may influence behaviours and habits around their sleep^8^.

Sleep has been recognised as an essential component in athlete preparation and is suggested to be one of the most effective recovery strategies available to athletes^5^. This is largely due to the regenerative and repair processes that occur to both the body and the brain during rapid eye movement (REM) and non-rapid eye movement (NREM) sleep^2^. Indeed, from a physical perspective, one of the important hormones responsible for muscle growth and repair, growth hormone, is mainly secreted at night, during NREM slow wave sleep^9,10^. Poor sleep also disrupts growth and repair of cells^10^ and individuals who sleep less may have impaired physical performance when compared to individuals who obtain a greater amount of sleep^11,12^. When the brain is concerned, sleep loss impairs cognition, learning and memory consolidation and mental well-being^13,14^.

Traditionally, REM sleep was considered more important for memory consolidation and mental processing, however, it is likely that a complex interaction of both REM and NREM is involved^15^. Furthermore, previous studies have found a high prevalence of obstructive sleep apnoea (OSA) and sleep-disordered breathing (SDB) in elite team and individual-sport populations, especially those in strength and power sports, highlighting that athletes may be susceptible to sleep disorders^16^. The prevalence of OSA is likely a consequence of large body mass and neck circumference in some sports (e.g., rugby union), which are anatomical factors that are consistent with this disorder^16^.

General sleep behaviours may be compromised in elite athletes due to a number of factors, including the increase in core temperature following exercise, increases in muscle tension, fatigue, and pain following training and competition, frequent international travel, disruption from light and noise, and increases in psychological stress^17^. Recent studies indicate that the amount of sleep an athlete obtains, as well as the sleep scheduling, might be dependent on the sport they participate in^18-21^. Supporting this, Lastella et al. (2015)^18^ suggested that athletes from individual-sports went to bed earlier and woke up earlier than athletes from team sports. Juliff et al. (2014)^6^ indicated that individual-sport athletes are similar to team-sport athletes in the reported occurrence of sleep complaints prior to major competitions. However, this is in contrast to the results reported by Erlacher et al. (2011)^22^, who observed greater reporting of poor sleep in individual-sport athletes compared with team-sport athletes. This difference was explained by the lower pressure and anxiety experienced in team sports as these athletes, unlike individual-sport athletes, are not solely responsible and accountable for their own results. In a study by Suppiah et al. (2021)^21^ evaluating sleep in 135 elite youth athletes, team-sport athletes obtained significantly worse Pittsburgh Sleep Quality Index (PSQI) global scores, sleep efficiency, and subjective sleep quality and had shorter sleep durations and later bedtimes than individual-sport athletes.

While the absolute differences in sleep quantity and quality and general sleeping patterns have been evaluated in individual and team-sport athletes, the reasons for some of these differences remain relatively unknown. Furthermore, to the authors knowledge, there are yet to be any studies that have evaluated the unique behavioural differences between team and individual sport athletes when it comes to their sleep habits. Gaining greater insight into these intricate differences may allow for targeted interventions to improve the sleep behaviours of athletes. Therefore, the current study aimed to identify differences in sleep habits and behaviours between individual and team-sport athletes using the Athlete Sleep Behavior Questionnaire (ASBQ)^17^ and the PSQI^23^.

## MATERIAL AND METHODS

In a cross-sectional design, a convenience sample of 407 elite athletes (137 individual-sport athletes [74 male/63 female], 270 team-sport athletes [163 male/107 female], mean±SD, age; 23±4y, Table 1) across 9 countries (Australia, Canada, England, India, Malaysia, New Zealand, Portugal, Sweden, USA) completed the two questionnaires via an electronic survey (Survey Monkey, Palo Alto Inc., CA, U.S.). Participants were recruited via a combination of social media channels, word of mouth and by targeting specific sporting organisations and national bodies (via email). The criteria for inclusion in the current study was: representation of their country at either national or international-level for their chosen sport, and a minimum of 4-weeks into the in-season phase of training for their sport. Athletes were surveyed from the following sports: individual-sports: badminton (n=5), boxing (n=7), cycling (n=30), golf (n=8), rowing (n=29), swimming (n=16), track and field (n=17), tennis (n=13), triathlon (n=12); and team-sports: basketball (n=16), football/soccer (n=15), cricket (n=17), hockey (n=34), netball (n=19), rugby league (n=31), rugby union (n=138). The study was approved by the Institutions Human Research Ethics Committee.

**Table 1:** Participant demographics. Data shown as means±SD.

	Team-sport athletes		Individual-sport athletes	
	Number	Age (y)	Number	Age (y)
Total	n=270	23 ± 4	n=137	21 ± 4
Male	n=163	24 ± 4	n=74	21 ± 4
Female	n=107	22 ± 4	n=63	22 ± 7

Once athletes met the inclusion criteria and opted into the study, both ASBQ and PSQI questionnaires were sent directly to the individuals via email in the same link. A full description of how to fill out each questionnaire was given, and athletes were asked to complete both questionnaires at the same time. The survey also included basic demographic information at the start (e.g., age, sex, sport, and country). According to the survey software, on average, completion of both questionnaires took 4.5 minutes. In line with the official instructions, both the ASBQ and PSQI ask participants to complete the questions relating to their normal sleeping habits and schedules over the previous 4 weeks. For this reason, athletes were required to be >4 weeks into the in-season phase of their training. All participants were cross-checked by researchers to ensure that they met the specified inclusion criteria.

The ASBQ is an 18-item survey that includes questions on sleeping behaviour and habits thought to be common areas of concern for elite athletes and has been used as a practical tool to identify maladaptive sleep behaviours^24^. The survey asks participants how frequently they engaged in specific behaviours over the past month (never, rarely, sometimes, frequently, or always). Weightings for each response (1=never, 2=rarely, 3=sometimes, 4=frequently, and 5=always) were summed to provide an ASBQ global score. A high score is indicative of poor sleep behaviours. The PSQI is a self-rated 19-item instrument intended to assess sleep quality and sleep disturbance over one month^23^. Scores range from 0 to 21, with higher scores indicating poorer overall sleep quality. Both the ASBQ and the PSQI have been shown to be valid and reliable tools for use in sleep monitoring^17,23,25^. These tools were selected because the ASBQ is the only questionnaire that identifies sleep behaviours specific to athletes, and the PSQI is one of the most widely used tools for measuring sleep quantity and quality in the general population^5^. Furthermore, while the PSQI might not be specific to athletes, it provides insight into important sleep scheduling factors and qualities such as bedtime, wake time, total sleep time (TST), sleep efficiency percentage (SE%), and sleep onset latency (SOL), which the ASBQ does not provide. Therefore, a combination of both questionnaires was implemented.

### Statistical analysis

Descriptive statistics are shown as means±SD unless stated otherwise. Statistical analysis was performed using SPSS V22.2 (IBM Corporation; Chicago, IL, U.S.). There were no outliers in the data, as assessed by inspection of a boxplot. Global scores for each questionnaire and each item of the ASBQ and PSQI were normally distributed, as assessed by Shapiro-Wilk’s test (*p*>0.05), and there was homogeneity of variances between groups, as assessed by Levene’s test for equality of variances (*p*>0.05). Comparison of individual and team athletes were performed for each questionnaire and each item of the ASBQ using independent samples t-tests, with statistical significance set at *p*<0.05. Cohen’s effect sizes (*d*) were calculated between individual and team-sport athletes for each questionnaire and interpreted using thresholds of 0.2, 0.5, 0.8 for *small, moderate* and *large*, respectively^26^.

## RESULTS

The self-reported TST in individual-sport athletes (7:57±0:55h:mm) was greater (*p*<0.05) than that reported by team-sport athletes (7:40±1:02h:mm, Table 2), without any significant differences in bed or wake times (*p*>0.05). These differences in TST were also associated with a significantly lower (*p*<0.01, *d*=0.29) SE% in team-sport athletes (88.6%) compared to individual-sport athletes (91.1%, Table 2).

**Table 2:** Key findings from the PSQI between team and individual-sport athletes, including *p*-values and effect-size comparisons between groups.

	Team (n=270)	Individual (n=137)	p-value	Effect-size (d)
Bedtime (pm)	22:31 ± 0:48	22:30 ± 0:53	0.80	0.03 *trivial*
Wake time (am)	7:16 ± 1:03	7:12 ± 1:07	0.62	0.06 *trivial*
Total sleep time (h:mm)	7:40 ± 1:02	7:57 ± 0:55	0.04*	**0.28 small**
Sleep efficiency (%)	88.6 ± 9.2	91.1 ± 7.4	0.009*	**0.29 small**
During the past month, how often have you had trouble sleeping because you…				
a) Cannot get to sleep within 30 mins	1.3 ± 1.0	1.2 ± 0.9	0.53	0.08 *trivial*
b) Wake up in the middle of the night or early in the morning	1.5 ± 1.0	1.0 ± 1.2	0.008*	**0.32 small**
c) Have to get up to use the bathroom	1.3 ± 1.0	1.0 ± 1.1	0.15	0.17 *trivial*
d) Cannot breathe comfortably	0.2 ± 0.6	0.2 ± 0.5	0.39	0.10 *trivial*
e) Cough or snore loudly	0.4 ± 0.8	0.3 ± 0.7	0.19	0.15 *trivial*
f) Feel too cold	0.6 ± 0.8	0.6 ± 0.8	0.97	0.01 *trivial*
g) Feel too hot	1.0 ± 1.0	0.7 ± 1.0	0.04*	**0.23 small**
h) Have bad dreams	0.6 ± 0.7	0.5 ± 0.7	0.19	0.16 *trivial*
i) Have pain	0.7 ± 0.8	0.7 ± 0.8	0.87	0.02 *trivial*
Sleep quality	1.1 ± 0.6	1.0 ± 0.7	0.38	0.11 *trivial*
**PSQI Global Score**	5.4 ± 2.8	5.2 ± 2.3	0.51	0.07 *trivial*

**Notes:** Data shown as means ± SD;

* Represents statistical significance (*p*<0.05).

There were no significant differences in PSQI global scores between team-sport and individual-sport athletes (Table 2). However, there was a difference between global scores for the ASBQ (Table 3) as well as multiple individual items from both questionnaires (*p*<0.05, Tables 2 and 3).

**Table 3:** Mean results for the ASBQ between team and individual-sport athletes including p-values and effect-size comparisons between groups.

Question	Team (n=270)	Individual (n=137)	p-value	Effect-size (d)
I take afternoon naps lasting two or more hours	1.9 ± 0.9	1.8 ± 0.9	0.14	0.16 *trivial*
I use stimulants when I train/compete (e.g., caffeine)	2.6 ± 1.3	2.1 ± 1.2	0.0001*	**0.38 small**
I exercise (train or compete) late at night (after 7 p.m.)	3.0 ± 1.1	2.9 ± 1.2	0.30	0.11 *trivial*
I consume alcohol within 4 hours of going to bed	2.0 ± 0.8	1.8 ± 0.8	0.005*	**0.30 small**
I go to bed at different times each night (more than ±1 hour variation)	2.8 ± 0.8	2.9 ± 0.8	0.61	0.05 *trivial*
I go to bed feeling thirsty	2.1 ± 0.8	2.0 ± 0.8	0.10	0.18 *trivial*
I go to bed with sore muscles	3.3 ± 0.7	3.1 ± 0.8	0.07	0.19 *trivial*
I use light-emitting technology in the hour leading up to bedtime (e.g., laptop, phone, television, and video games)	4.1 ± 0.9	4.3 ± 0.8	0.10	0.16 *trivial*
I think, plan and worry about my sporting performance when I am in bed	3.0 ± 0.9	2.9 ± 1.0	0.31	0.11 *trivial*
I think, plan and worry about issues not related to my sport when I am in bed	3.0 ± 0.9	3.0 ± 0.9	0.99	0.00 *trivial*
I use sleeping pills/tablets to help me sleep	1.3 ± 0.6	1.2 ± 0.6	0.35	0.10 *trivial*
I wake to go to the bathroom more than once per night	2.0 ± 0.9	1.9 ± 0.8	0.08	0.18 *trivial*
I wake myself and/or my bed partner with my snoring	1.5 ± 0.8	1.4 ± 0.9	0.44	0.08 *trivial*
I wake myself and/or my bed partner with my muscle twitching	1.9 ± 0.9	1.9 ± 1.0	0.59	0.06 *trivial*
I get up at different times each morning (more than ±1 hour variation)	2.7 ± 0.9	2.8 ± 1.0	0.34	0.11 *trivial*
At home, I sleep in a less than ideal environment (e.g., too light, too noisy, uncomfortable bed/pillow, too hot/cold)	2.0 ± 0.9	1.9 ± 0.8	0.22	0.12 *trivial*
I sleep in foreign environments (e.g., hotel rooms)	2.6 ± 0.9	2.5 ± 0.8	0.18	0.14 *trivial*
Travel gets in the way of building a consistent sleep-wake routine	2.4 ± 0.9	2.2 ± 0.9	0.03*	**0.24 small**
**ASBQ GLOBAL SCORE**	44.3 ± 5.9	42.6 ± 5.7	0.004	**0.30 small**

**Notes:** Data shown as means ± SD;

* Represents statistical significance (*p*<0.05).

## DISCUSSION

The current study found *small*, but significantly greater self-reported TST in individual-sport athletes compared to team-sport athletes, without any significant differences in bed or wake times. The differences in TST were associated with a significantly lower SE% in team-sport athletes (88.6%) compared to individual-sport athletes (91.1%). These differences in TST and SE% were also associated with sleep behaviours and habits, where individual athletes generally reported better sleep behaviours, as identified via global scores for the ASBQ and individual items in the PSQI.

The results in the current study are in contrast to previous research by Erlacher et al. (2011)^22^, who observed a higher incidence of poor sleep in individual-sport athletes compared with team-sport athletes. However, in the Erlacher et al.^22^ study, athletes were surveyed on their sleep behaviours before competition, with researchers postulating that individual athletes may be more susceptible to poor sleep due to higher levels of anxiety^27^ and that team sport athletes are more likely to play week-to-week and have better routines and coping strategies compared to the irregular nature of competiton in individual-sports. Our study intended to evaluate general sleeping habits and behaviours over a 4-week period, rather than around specific competitions or events. Interestingly, the results in our study are also in contrast to the findings from Lastella et al. (2015)^18^ who reported that athletes from individual sports went to bed earlier, woke up earlier and obtained less sleep (~6.5 hours) than athletes from team sports (~7 hours). The self-reported TST in the current study was 7h:40mins for team sport athletes and 7h:57mins for individual sport athletes. While the current study used self-reported TST via the PSQI, and the Lastella et al.^18^ study determined TST using objective measures (wrist activity monitors), it is still interesting that these results were in direct contrast to each other.

It is therefore likely that even with individual and team sport classifications, there are many different scheduling and behavioural factors involved. For example, the individual sport athletes in the Lastella et al.^18^ study were from pre-dominantly endurance-based sports such as swimming, cycling, triathlon, and race-walking. Many of these sports traditionally include early-morning training and high training volumes and durations, whereas many of the individual sports in the current study might not necessarily be associated with early or late-night training (e.g., badminton, golf, and tennis). Conversely, our results are in agreement with the findings of Suppiah et al. (2021)^21^, who observed better sleep characteristics in elite individual-sport youth athletes compared to those in team-sports. Indeed, the individual sport cohort in the Suppiah et al.^21^ study also included many non-endurance based individual athletes. The inconsistencies in athlete sleep characteristics by sport type between studies highlights that contextual between-sport differences may exist. These potential between-sport differences in athlete sleep characteristics warrant further investigation and highlight the possibility that instead of comparing individual vs. team sport athletes, perhaps a more appropriate comparison may be between endurance-based vs. strength/power-based athletes.

While there were no significant differences between team and individual-sport athletes for the PSQI global score, there were significant differences in individual questionnaire items. Team-sport athletes reported significantly more (*p*<0.05) occurrences of “*waking in the middle of the night or early in the morning*” and “*feeling too hot*”, both associated with *small* effect size (*d*=0.23-0.32) differences between groups. Training or competing in the evening has been linked to increases in core temperature^2^. We speculate that the difference between groups for the “*feeling too hot*” item of the questionnaire may be because the team-sport athletes in the studied cohort were more likely to train and compete in the evening, compared to the individual-sport athletes (e.g., rowing, swimming, and triathlon).

For the ASBQ, there was a significantly higher global score for team-sport athletes (*p*<0.01) when compared to individual-sport athletes (44.3 vs. 42.6, respectively), indicating poorer sleep-behaviours in the team-sport athletes. Three individual questionnaire items showed *small* but significant differences between team and individual athletes, with team-sport athletes reporting higher occurrences for all three. These items were “*I use stimulants when I train/compete (e.g., caffeine)*”, “*I consume alcohol within 4 hours of going to bed*”, and “*Travel gets in the way of building a consistent sleep-wake routine*”. These findings also highlight the importance of administering sleep instruments that elicit themes from environmental, behavioural, and sport-related factors to understand athletes’ distinct sleep characteristics and may allow for the implementation of targeted interventional strategies.

Given both individual and team-sport athletes reported obtaining less than the recommended 8 hours of sleep per night, the current study confirms that of previous studies^16,18^, suggesting that athletes may struggle to get adequate amounts of sleep. This is of concern, as previous research shows that poor sleep may be impairing cognition, learning, and memory consolidation, mental well-being^13,14^, and performance^11,12^ in athletes. When considering the group as a whole in the current study (team and individual athletes), the main areas of concern relating to sleep behaviours were around technology/device use at night, going to bed with sore muscles, and lying awake worrying about both sport and non-sport related issues, all of which can be addressed via targeted interventions.

Further methods and strategies of improving sleep quantity and quality in athletes are therefore needed. Targeting maladaptive sleep behaviours may allow practitioners and researchers to use specific interventions that help athletes to change their sleep habits. The ASBQ has been used previously by targeting the individual items on the questionnaire where athletes indicate “sometimes”, “frequently” or “always” with specific behaviour modifications related to those items^24^. This resulted in significant improvements in SE% (+5%), SOL (-29 minutes), and sleep onset variance (-28 minutes) in elite cricket athletes^24^.

In conclusion, this is the first study to delve into the unique sleep habits and behaviours of team and individual-sport athletes, in a large cohort of elite athletes (n=407). On average, team-sport athletes displayed poorer perceived sleep quantity and quality than individual-sport athletes, which was also associated with poorer overall sleep behaviours. The main behavioural factors associated with these differences were related to the use of supplements (e.g., caffeine), consuming alcohol, and regular travel interrupting sleep/wake routines. Team-sport athletes also reported waking up more during the night and had a greater occurrence of feeling too hot when trying to fall asleep. Common concerns amongst the entire cohort of athletes related to technology/device use at night, going to bed with sore muscles, and lying awake worrying about both sport and non-sport related issues. Where common sleep issues are present in certain sports, we would recommend a broader conversation amongst coaches, practitioners, and administrators to identify ways (e.g., training scheduling, travel, and recovery strategies) whereby widespread changes can be made to benefit the health, well-being, and performance of the athlete.
